# Electron–phonon interaction toward engineering carrier mobility of periodic edge structured graphene nanoribbons

**DOI:** 10.1038/s41598-023-32655-9

**Published:** 2023-04-08

**Authors:** Teng-Chin Hsu, Bi-Xian Wu, Rong-Teng Lin, Chia-Jen Chien, Chien-Yu Yeh, Tzu-Hsuan Chang

**Affiliations:** 1 Graduate Institute of Electronics Engineering (GIEE), National Taiwan University, Taipei, Taiwan; 2 Department of Materials Science and Engineering, National Taiwan University, Taipei, Taiwan; 3 Department of Electrical Engineering, National Taiwan University, Taipei, Taiwan

**Keywords:** Electrical and electronic engineering, Electronic properties and devices, Synthesis of graphene

## Abstract

Graphene nanoribbons have many extraordinary electrical properties and are the candidates for semiconductor industry. In this research, we propose a design of Coved GNRs with periodic structure ranged from 4 to 8 nm or more, of which the size is within practical feature sizes by advanced lithography tools. The carrier transport properties of Coved GNRs with the periodic coved shape are designed to break the localized electronic state and reducing electron–phonon scattering. In this way, the mobility of Coved GNRs can be enhanced by orders compared with the zigzag GNRs in same width. Moreover, in contrast to occasional zero bandgap transition of armchair and zigzag GNRs without precision control in atomic level, the Coved GNRs with periodic edge structures can exclude the zero bandgap conditions, which makes practical the mass production process. The designed Coved-GNRs is fabricated over the Germanium (110) substrate where the graphene can be prepared in the single-crystalline and single-oriented formants and the edge of GNRs is later repaired under "balanced condition growth" and we demonstrate that the propose coved structures are compatible to current fabrication facility.

## Introduction

The technology to open bandgap of graphene nanoribbon (GNR) while maintaining its high mobility for mass production under wafer-scale production has been desired in semiconductor manufacturing industries for years^1–4^. GNRs can offer a controllable band gap that meet the different demands in transistors^5,6^, photovoltaics^7^, spin electronics^8–10^ and quantum devices^11^. The controllable band gap of GNRs can be opened through quantum confinement, which depends both on the width and edge structure of the ribbons^12–14^. The GNRs with the quantum confined edge, armchair-edged GNRs (AGNRs) or zigzag-edged GNRs (ZGNRs), have opened bandgap as the widths of GNRs narrow down to few nanometers^13,15–17^. While the bandgap of GNRs with quantum confined edge can be increased through the width narrowness, it comes at the cost of mobility reduction^18–20^. Moreover, with only a few atoms varied, the bandgap of AGNRs, as well as ZGNRs, can be reduced to zero^21–23^. This makes it challenging to generate wafer-scale, bandgap opened, and high performance GNRs. Therefore, a fabrication method of large-scale GNRs to have high mobility without the penalty of bandgap losses is needed.

Two main methods have been established to prepare GNRs, grouped by ‘bottom-up’ approaches^24^ and ‘top-down’ approaches^25,26^. The bottom-up approaches were based on aggregating small segments of precursor/seed into stripes leading to the successful synthesis of GNRs with accurate edge structure and sub-nanometer width^27–29^. Recently, studies have simultaneously improved the band gap and mobility of GNRs through constructing the periodic edge structures produced by bottom-up approaches. Introducing the curved^30^ or fjord-type^31^ periphery on the edge of GNRs can significantly alter their topological conformations and electronic structures. Furthermore, it has been shown the theoretical and experimental possibility to further increase the carrier mobility by several orders while maintaining the bandgap conditions via special designs for the periodic edge profile of GNRs^29^. With measuring electrical properties of structured GNRs, the carrier mobility and optical bandgap of GNRs were estimated to be 150–15,000 cm^2^ V^−1^ s^−1^ and 1.88 eV respectively, which is close to the calculated mobility (18,700 cm^2^ V^−1^ s^−1^) and bandgap (2.056 eV)^27,32^. Therefore, the main challenge of GNRs mass production is to achieve the wafer scale of designed structured GNRs.

In addition to bottom-up strategies, the top-down approaches were more compatible to the semiconductor industries^33,34^. These top-down approaches were based on graphene as substrate initially and then pattern into the ribbons by subtractive preparing (e.g., full lithography and etching covering)^35,36^. Besides, the lithography fabrication can also be used to define GNRs with periodic geometry^34^. Recently, top-down generated GNRs can be further refined in its edge profile and be recomposited from defective edges to zigzag edge or armchair edges via the union of both top-down and bottom-up approaches^37^. This methodology can compensate for the scalability of location and geometry in traditional bottom-up approaches to achieve the GNRs array. This method has lightened the path to generate wafer scale GNRs with consistent edge geometry.

**Figure 1:**
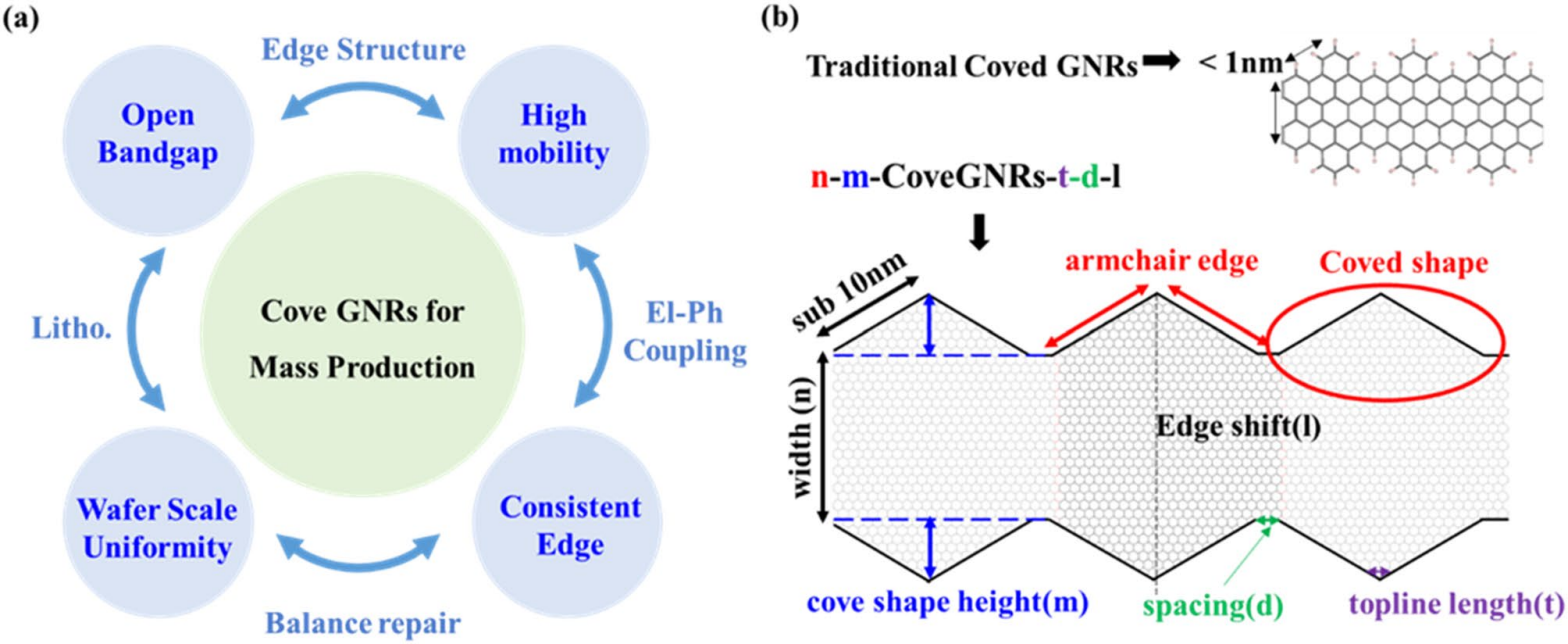
(**a**) Designed structure of CoveGNRs to solve the main issue in the mass production of GNRs. (**b**) Dimension scale of width and edge structure in traditional CoveGNRs is less than 1 nm. Dimension scale of our designed CoveGNRs is about 4–14 nm that can be fabricated in lithography to reach wafer scale.

In this paper, we proposed a design of CoveGNRs with large periodic structure and demonstrate a scalable and precisely controlling nanoribbons strategy to fabricate CoveGNRs, as illustrated in Fig. 1a. Our periodic cove structure are designed with range from 4 to 14 nm or more, which can be fabricated by advanced lithography tools, such as Helium Ion Beam Lithography (HIBL)^38^ and Extreme Ultra-Violet Lithography (EUVL)^39^. The proposed CoveGNRs can exclude the zero bandgap conditions in AGNRS when the number of atoms along the width equals to 3N + 2^12,21,28^. By suppressing electron–phonon scattering and breaking the localized electronic state that limit the electronic performance, the mobility of CoveGNRs can be enhanced several orders compared with the traditional ZGNRs in the same width while having bandgap. The feasibility of the designed structure was demonstrated with the fabrication process. The CoveGNRs are fabricated through wafer-scale graphene growth on Ge (110) substrate. The crystalline and accuracy of the edge of CoveGNRs can be regulated by controlling the equilibrium growth approaches^40^. The edge of patterned nanoribbons is repaired with "balanced regrowth condition" that is tuned with deposition rate of graphene grains close to zero to enable the process of repairing the damaged edge during deposition and etching process^33^. This method can repair defective edges to improve the electrical properties of GNRs because it is highly dependent on the edge quality of the materials.

## Methodology

### Design of cove-structure in GNRs

In order to do a qualitative analysis in band gap and mobility of CoveGNRs compared with ZGNRs, we used the density functional theory (DFT) to calculate electron structure of GNRs and Density functional perturbation theory (DFPT) to calculate the vibration properties and electron–phonon(el–ph) coupling in consideration of the full electron band and phonon dispersion. All the DFT calculations were based on the QUANTUM-ESPRESSO package^41^. The unit cell used in the package are all 3D crystal lattice with the vacuum layer set to 15 Å in order to eliminate interlayer interaction of GNRs in 3D unit cell. 15 Å separation is enough to cut off the overlap between the wavefunction. With vacuum layer causing the edge stress on GNRs, the bonding length of whole GNRs will be distorted to reach the equilibrium state. To calculate band gap and phonon dispersion of GNRs under equilibrium state, the lattice cell and atomic site were self-relaxed and the total force of GNRs are required to converge to less than 1E−5 Ry/bohr in the DFT calculation. To get band gap on accurate high symmetric point, a 36 k-points in monk horst sampling with uniformly positioned along the 1D Brillouin zone were used.

Since the band gap calculation of the designed GNRs in this work is time-consuming in DFT, we employed the tight-binding model^29^ to study the GNRs extracted onsite energy and hopping energy with DFT to be 0 eV (the energy of 2pz is set to be zero) and 3.033 eV. The value extracted from is agreed with theoretical calculation^42^ and measurements of angle resolved photoemission spectra (ARPES)^28,43,44^.

To estimate the mobility of the GNRs, the el–ph coupling of structured GNRs is needed to be considered^18^. With the linear relation of charge density and potential to the el–ph coupling, phonon coupling matrix elements with the scattering of an electron at band *i* with wavevector *k* to another state at band *j* with wavevector *k* + *q* over a phonon mode λ in wavevector *q* can be written as

1 $${g}_{ji}^{\lambda }\left(k,q\right)= \sqrt{\frac{\hslash }{2M{\omega }_{q\lambda }}}\langle {\psi }_{jk+q}|{\Delta }_{q\lambda }{V}_{SCF}|{\psi }_{ik}\rangle$$

where ${\psi }_{ik}$ and ${\psi }_{jk+q}$ are the wavefunction of electron and ${\Delta }_{q\lambda }{V}_{SCF}$ is the derivative of electron potential under atomic displacement associate over a phonon λ in wavevector *q.* Under the relaxation time approximation in different interaction time, the el–ph scattering relaxation time ${s}_{i}^{\lambda }(k,q)$ is given by^45^

2 $${s}_{ik}={\sum }_{q\lambda }{s}_{i}^{\lambda }(k,q)= \frac{2\pi }{\hslash }\sum_{q\lambda j}{\left|{g}_{ji}^{\lambda }(k,q)\right|}^{2}\times \left\{\left.\left[{f}_{jk+q}+{n}_{q\lambda }\right]\delta \left({\varepsilon }_{jk+q}-{\varepsilon }_{ik}-\hslash {\omega }_{q\lambda }\right)+\left[1+{n}_{q\lambda }-{f}_{jk+q}\right]\delta ({\varepsilon }_{jk+q}-{\varepsilon }_{ik}+\hslash {\omega }_{q\lambda }\right)\right\}$$

where ${f}_{jk+q}$ is the electron equilibrium distribution that is Fermi–Dirac distribution and ${n}_{q\lambda }$ is the phonon Bose–Einstein distribution. The first and second *δ*-functions describe the absorption and emission of a phonon, respectively. The charge mobility can be obtained by solving the quantum Boltzmann transport equation^46^ in the relaxation time approximation to the first order at the weak electric field. The low field carrier mobility with fermi velocity ${v}_{ik}$ = 1/ℏ ∇${\varepsilon }_{ik}$ can be described as

3 $$\mu =e\frac{\sum_{i}\int (1/{s}_{ik}){{v}_{ik}}^{2}\frac{\partial {f}_{ik}}{\partial {\varepsilon }_{ik}}dk}{\sum_{i}{f}_{ik}dk}$$

### Preparation of CoveGNRs

Ge (110) substrate was placed into the quartz chamber heater with three-zone to precisely control the temperature in process of chemical vapor deposition (CVD)^31^. Before the deposition, the chamber was evacuated to 1E−6 Torr with turbo pump for four hours and was re-filled back to 1 a.t.m. with 200 s.c.c.m Ar and 100 s.c.c.m H_2_. After the environment of the chamber was stabilized at ambient, the furnace was sequentially heated up from 500 to 910 °C gradually without the spike of the temperature; The sample was held under annealing with flow of 200 s.c.c.m. Ar and 100 s.c.c.m. H_2_ for 2 h and later with the addition of the 3.3 s.c.c.m. CH_4_ for 12 h for full coverage deposition of graphene. After the deposition, standard e-beam lithography was applied in patterning the array of GNRs with two structures: the double-side wavy structures with the spacing of 60 nm and the 200 nm diameter Graphene dots. A mild O2 plasma was applied to the sample to define the graphene structures (power: 50 W, pressure: 10 mTorr, flow rate: 50 s.c.c.m., time: 8 s). The Graphene/Ge (110) sample was then placed back in the growth chamber for post-annealing process for 120 min i to lower the carbon contamination. The chamber was then evacuated and refill back the ambient condition to further repair the patterned GNRs/Ge (110) with 200 s.c.c.m. Ar, 100 s.c.c.m H_2_, and a lowered flow rate of CH_4_ for 120 min, 0–1.5 s.c.c.m., and cooling down to ambient temperature under the constant flow of 200 s.c.c.m. Ar, and 100 s.c.c.m H_2_.

## Result and discussion

### Stable opening band gap in CoveGNRs

In this study, we only focused on designed CoveGNRs built from ZGNRs, which was feasibly fabricated by trimming graphene into GNRs along zigzag direction and repaired its edge roughness. Traditional CoveGNRs, which was shown in the upper part of Fig. 1b, built from ZGNRs introduces partial armchair character that show high mobility^27^ and low bandgap^47^. In order to achieve opened-bandgap GNRs in wafer scale fabrication, the GNRs designed in this paper, which was shown in the lower part of Fig. 1b, contained periodic edge structures with features from a few up to 20 times ‘cove’ edge structures. This feature size of the cove structures is in height about 3 nm and the side length about 6 nm with apex angle in 120° which can be fabricated in advanced lithography tools. To ensure the CoveGNRs with opened bandgap, the mechanism between bandgap and geometry are analyzed through two parameters including the width of the ribbon (*n*) and the number of atoms of cove shape in height (*m*) are used to define the Cove GNRs.

Besides, the defect edge or random edge hamper opening bandgap of GNRs with quantum confined edge. Hence, a designed edge structure of GNRs is needed toward defect endurable band gap and structure compatible to edge repairing fabrication. The randomness of edge profile in our designed CoveGNRs created during the fabrication are characterized through four parameters: *d, t, l, θ.* The design of the cove structures to maintain electrical properties of GNRs with the edge randomness in wafer-scale fabrication will be focused. With considering the variation about geometry and randomness of edge profile, we can analysis the dynamic edge profile of CoveGNRs in repairing process and the influence of edge profile to the bandgap. For instance, the zigzag in the spacing between the cove shape (*d*), the zigzag in the topline of cove shape (*t*), and the angle variation between the orientation of cove edge and the orientation of armchair edge (*θ*) can describe the major randomness between the design and the preparation of the GNRs. The edge shifting between above and below cove shape (*l*) in the lithography process are needed to consider as well.

**Figure 2:**
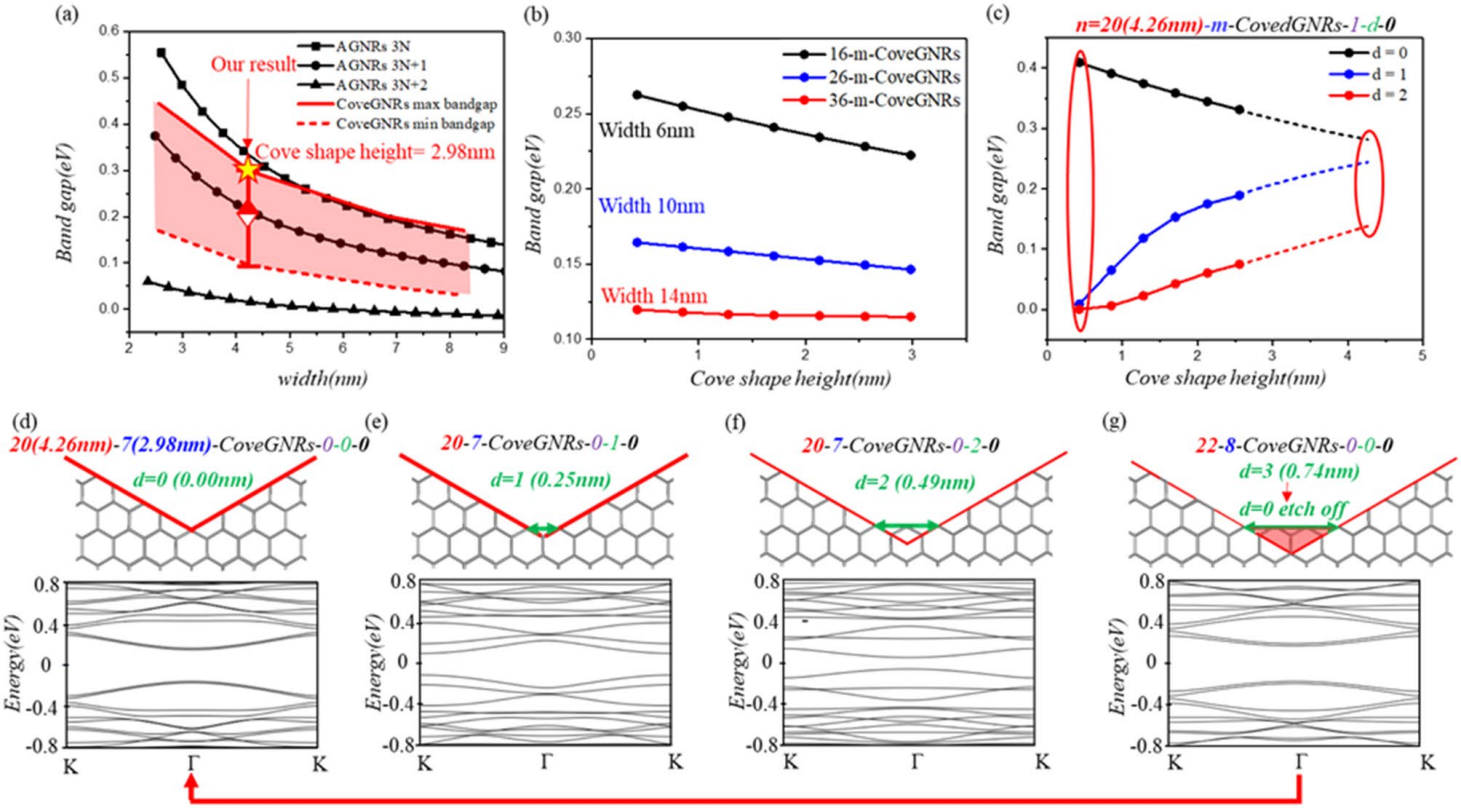
(**a**) Bandgap variation as function of the width in CoveGNRs compare with the AGNRs. (**b**) Bandgap variation of CoveGNRs in different cove shape size (label in the number of atoms along the height of cove shape) in case the width 6, 10, 14 nm. (**c**) Bandgap variation versus zigzag edge amount (*d*) in the spacing of cove shape size of Cove GNRs. The cove shape of CoveGNRs increasing can eliminate the zero band gap caused by zigzag edge segment of Cove GNRs in the anisotropic annealing. (**d**–**g**) The spacing between the cove structure with zigzag edge segment. As the zigzag edge segment increasing from *d* = 0 to *d* = 2, the zigzag edge can be etched into armchair edge.

The variations of bandgap in CoveGNRs with edge randomness case (*d, t, l, θ*) as function of the width are summarized in Fig. 2a. The width of GNRs will determine the distribution of wavefunction and affect the bandgap of GNRs be negative exponential proportion to its width^48^. The bandgap of ANGRs have three negative exponential trends as function of width correspond to the number of atoms along the width equals 3N, 3N + 1, 3N + 2 (N is integer). In the case of 3N + 2, the bandgap of AGNRs will approach to zero when width scales up to 10 nms. This drawback can be alleviated by the cove structure of CoveGNRs. By manipulating edge structure to affect the boundary condition of wavefunction^49^, our designed CoveGNRs can exclude the zero-bandgap transition in 3N + 2 rule of AGNRs and still maintain 0.1 eV bandgap in width 4.5 nm, which is within the height of cove shape about 3 nm. The CoveGNRs without edge randomness case (*d, t, l, θ*) have maximum bandgap and it has been found that the spacing *d*, however, plays a key role in determining the minimum bandgap of the CoveGNRs. The individual influence of bandgap cause by the other parameters *t, l, θ* are introduced in Supp. Figs. S8–S10.

As shown in Fig. 2b, we explore the relation between the bandgap of CoveGNRs and its geometry, defined in parameter *n* and *m*. Without considering the randomness of edge profile, the bandgap of the CoveGNRs and the height of cove shape (*m*) are in negative correlation. The bandgap of CoveGNRs decreases as *m* increase, and this reduction is lesser when the width (*n*) of GNRs is broader. In the case of CoveGNRs in 14 nm width, the decline of bandgap is less than 0.1 eV when *m* increases from 0.25 to 3 nm. This CoveGNRs, with *n* = 14 nm and *m* = 3 nm, can have opened bandgap of which is stable to the variation of width and cove structure. This feature bridges the gap between GNRs fabrication and critical dimension required for mass production.

Among the parameters in featuring edge randomness to be considered, the amount of zigzag segment between the spacing (*d*), play a key role in determining the bandgap of the GNRs. The formation mechanism of this zigzag segment is due to the fact there is no enough space to form the armchair edge between spacing of cove structure when *d* equal to 0–2. When *d* equal to 3, the zigzag segment can be etched off to form armchair edge like in case *d* equal to 0. Such structure transformation can be illustrated in Fig. 2d–g. In addition, since different amounts of zigzag segments will change the boundary condition of the wavefunction in CoveGNRs, the band structures of different *d* are non-similar and lead to change in the bandgap. Even though, three trend of band gap in CoveGNRs as a function of cove shape height can be observed Fig. 2c. In the case of CoveGNRs in width 4.26 nm, the band gap becomes close to 0 in the case *d* equal to 1, 2 when the height of cove shape is smaller than 1 nm. This zero-bandgap condition can be eliminated when the cove shape becomes bigger to reach the lithography availability. In the case of zigzag segment in the topline of cove shape, the band gap variation of CoveGNRs as function of *d* is similar to the case of *t*. This is very similar variation of these two situations and it shows zigzag edge concentration is harmful to open the bandgap of CoveGNRs. We can see the smallest bandgap of spacing (*d*) = 0.49 nm, minimum width (*n*) = 4.26 nm in CoveGNRs still have 0.08 eV. Since the anisotropic repairing of balance repair condition can achieve consist armchair edge of graphene, we propose that we can succeed in almost no zigzag segment of CoveGNRs (*d* = 0).

The band gap of CoveGNRs under the variation of topline and spacing cove shape is converged and have less oscillation and compare to the band gap of AGNRs case. With this distinguished band gap characteristic of CoveGNRs, the gapless case can be avoided through controlling the cove shape size although it contains zigzag in the topline or the spacing of the cove shape. By increasing the size of the cove shape to alleviate and close the variation of the band gap between the defective edge case and without defective edge case. That can realize the promotion of CoveGNRs to mass production without controlling the precise scale of the atomic level. The stability of the band gap can be improved in the scale of semiconductor fabrication by rescaling of cove shape.

### Engineering the electron–phonon scattering of CoveGNRs

**Figure 3:**
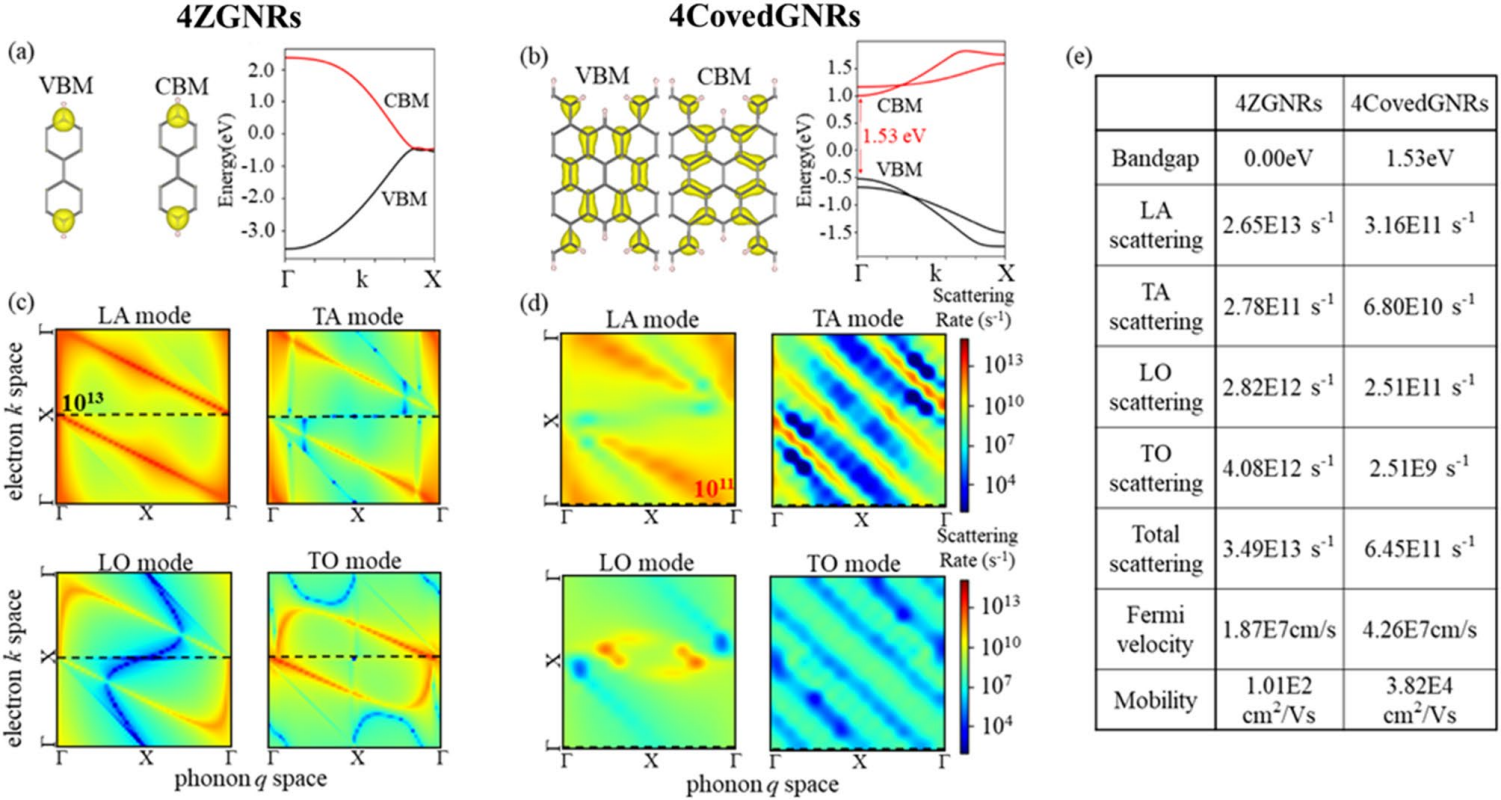
Electronic properties of 4ZGNRs and 4CoveGNRs, respectively. (**a**,**b**) The electron density distribution of CBM/VBM in unit cell and their band structure along the high symmetry lines. (**c**,**d**) The full band calculation of electron–phonons scattering rate at conduction band. The dash line highlights the CBM and is integrated to calculate the scattering time at CBM. (**e**) Band gap, individual scattering rate in LA/TA/LO/TO phonon mode, total scattering rate, fermi velocity and mobility value of 4ZGNRs and 4CoveGNRs.

In this work, the Boltzmann transport equation with the el–ph scattering in fermi golden rule is used to calculate the mobility. Instead of extracting the mobility through effective mass approximation, the Boltzmann transport equation can avoid the inaccuracy of mobility estimation in the cases of linear energy–momentum relation in AGNRs and ZGNRs that cannot be approximated properly^20^. In carrier transport of GNRs, the mobility will be influence by the carrier density, fermi velocity and scattering time. The valence band maximum (VBM) and conduction band minimum (CBM) in band diagram has the maximum carrier density. In Fig. 3a,b, the CBM/VBM are at X point in 4ZGNRs and Γ point in 4CoveGNR, respectively. We engineered the lattice constant and structure of 4CoveGNRs to fold the CBM/VBM at X point in 4ZGNRs into Γ point in 4CoveGNR, as shown in Supp. Fig. S3. The scattering event, e.g. el–ph scattering, electron–defect scattering and surface scattering, will hinder the electron transmission and lead to shorten the scattering time. Based on the Matthiessen's rule^41^, the total scattering time can be calculated by the harmonic mean of individual scattering times and the minimum scattering times will be the determinant term of total scattering. In the case of intrinsic carrier transport of GNRs without doping, we calculated the el–ph scattering with the minimum scattering times to estimate the mobility. Figure 3c,d depict the scattering time *τ(i,k,λ,q)* as function of electron *k* space in conduction band and phonon *q* space in different mode LA, TA, LO, TO that derived from the el–ph coupling matrix by Eq. (1) of 4ZGNRs and 4CoveGNRs, respectively. The total el–ph scattering is combined by the scattering in different phonon mode and the scattering with minimum scattering time will be the determinant term in total scattering time. In the case of 4ZGNRs, the scattering time has the lowest value at the Γ point in q space in case of LA, TA, LO, TO phonon mode. The scattering time for 4ZGNRs in Γ point of LA phonon mode is the lowest value in all phonon mode and is about 1E−14 s that will be the determinant term in total el–ph scattering. In the case of 4CoveGNRs, the determinant scattering time is about 1E−12 s near Γ point of LA phonon mode, which is two order higher than the case of 4ZGNRs. The scattering time is strongly related to the spatial distribution of its wavefunction in real space. Previous studies have shown that wavefunction running along the stretching direction of the ribbon generally results in longer scattering time^20^ and this results that the total scattering time of 4CoveGNRs is larger than 4ZGNRs.

Besides the scattering time in el–ph coupling, the fermi velocity is the dominant terms of mobility in Boltzman transport equation. The fermi velocity at CBM of 4CoveGNRs is two times bigger than 4ZGNRs, which are listed in Fig. 3e. The additive two cove structure of 4CoveGNRs greatly changes the distribution of the wave function. While in the case of 4CoveGNRs, the CBM and VBM are completely delocalized. The carrier transport mobility is generally proportional to the spatial area of charge carriers^50^, with more delocalized carrier providing higher fermi velocity. After calculated the scattering time and fermi velocity, we can apply the quantum Boltzmann transport equation in Eq. (2) to calculate the carrier mobility.

### Engineering the electron–phonon scattering to enhance mobility of Cove GNRs

We use quantum BTE in Eq. (1) to calculate mobility. Three variables in equation of quantum BTE give determinant influence, fermi velocity, electron Fermi–Dirac distribution and scattering rate. All of variable are calculate in the reciprocal k state of the ith band and integrate over in full Brillouin zone. Derivative of carrier density will give an maximum value at CBM/VBM. Fermi velocity ${v}_{ik}$ = 1/ℏ ∇${\varepsilon }_{ik}$ around CBM/VBM of 4ZGNRs and 4CoveGNRs is 1.87E7 cm/s and 4.26E7 cm/s, respectively.

The scattering event, e.g. el–ph scattering, electron–defect scattering and surface scattering, will hinder the electron transmission and raise the scattering rate. Based on the Matthiessen's rule^41^, the total scattering rate can be calculated by the sum of individual scattering rate and the maximum scattering rate will be the determinant term of total scattering. In the case of intrinsic carrier transport of GNRs without doping, we calculated the el–ph scattering with the maximum scattering rate to estimate the mobility. Figure 3c,d depict the scattering rate *s*(i,k,λ,q) as function of electron k space in conduction band and phonon q space in different mode LA, TA, LO, TO that derived from the el–ph coupling matrix by Eq. (2) of 4ZGNRs and 4CoveGNRs respectively. The total el–ph scattering is combined by the scattering in different phonon mode and the scattering with maximum scattering rate will be the determinant term in total scattering rate.

The el–ph coupling matrix can be calculated by Eq. (3). The bra-ket in Eq. (3) are the wavefunction of electron at j band and i band of the k + q state and k state respectively. Since the ${n}_{q\lambda }$ phonon Bose–Einstein distribution and ${f}_{ik}$ electron Fermi–Dirac distribution will give a maximum value of el–ph scattering rate in calculating the electron mobility, we only need to consider the el–ph coupling matrix at q = 0 and k at CBM. Therefore, the wavefunction of electron at CBM plays a determinant influence of mobility. The additive two cove structure of 4CoveGNRs greatly changes the distribution of the wave function. Rather than the wavefunction in CBM/VBM of 4ZGNRs is localized in the edge, the CBM/VBM of 4CoveGNRs are completely delocalized. In calculating the bra-ket of el–ph coupling matrix, the delocalized wavefunction gives smaller el–ph coupling matrix, and results shorter scattering rate and faster mobility. Previous reference also shows that the carrier transport mobility is generally proportional to the spatial area of charge carriers^40^, with more delocalized carrier providing lower scattering rate. After calculated the scattering time and fermi velocity, we can apply the quantum Boltzmann transport equation in Eq. (2) to calculate the carrier mobility. As shown in Fig. 3e, The electron mobility at 300 K of CoveGNRs is 4.15E4 cm^2^/Vs, which is two orders higher than the ZGNRs (about 1.01E2 cm^2^/Vs).

### Preparation of the CoveGNRs on Ge(110) substrate

**Figure 4:**
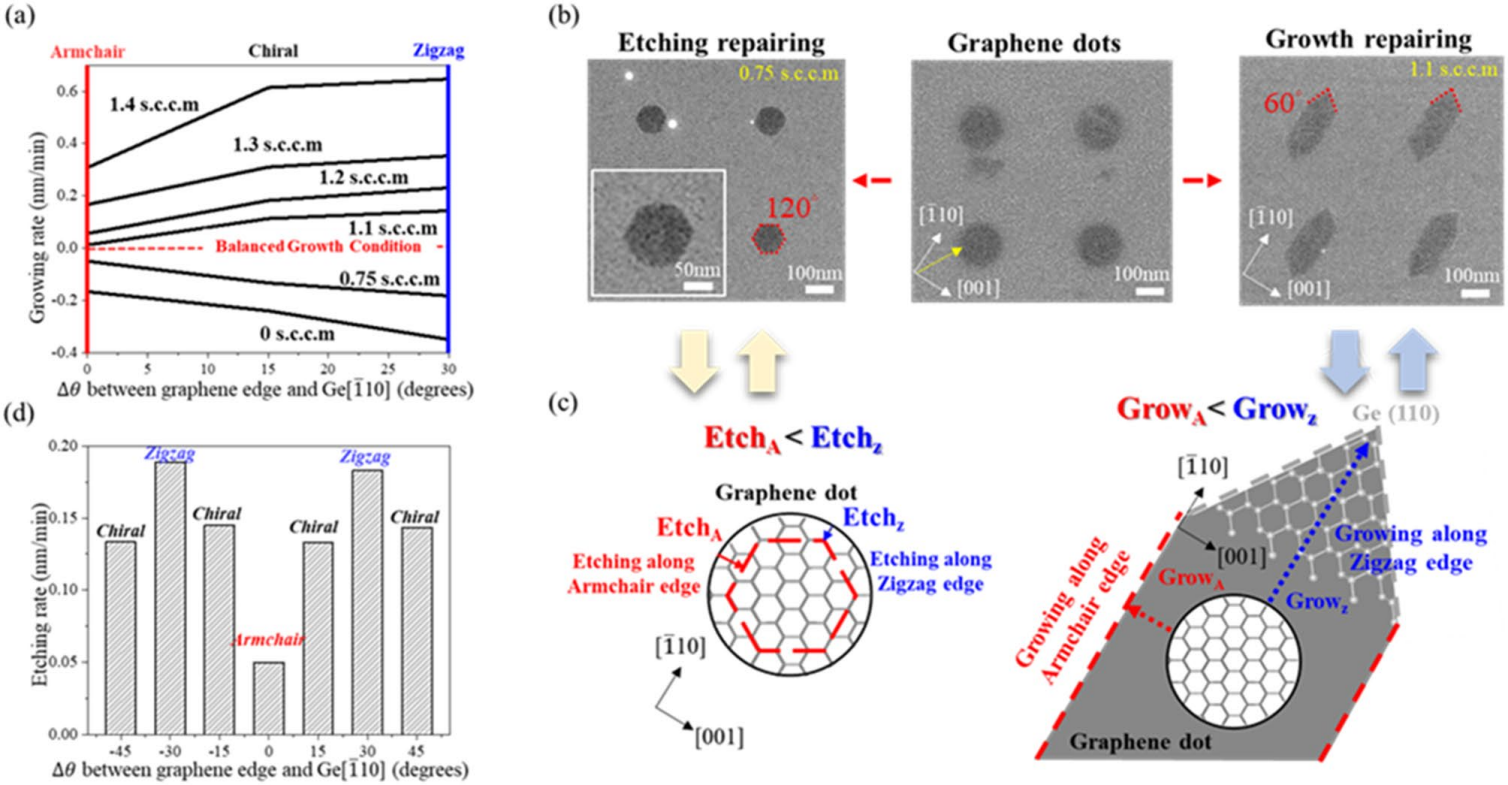
(**a**) Growth rate of the GNRs along with different orientations along Ge[110] under 1.4 s.c.c.m., 1.3 s.c.c.m., 1.2 s.c.c.m., 1.1 s.c.c.m., 0.75 s.c.c.m., and 0 s.c.c.m. CH4 conditions. (**b**) SEM images after regrowth the graphene dots with 1.1 s.c.c.m. and 0.75 s.c.c.m. CH4 precursor. (**c**) The schematic diagram of the etching and growth of graphene dots along with armchair and zigzag edges. (**d**) Etching rate of the GNRs along with different angles under 0 s.c.c.m. CH4 condition.

To control the edge profile of the patterned GNRs, the graphene dots samples was subjected to the regrowth process under the different CH_4_ flow rates. In terms of the Wulff Construction (KWC) theory^51,52^, which was widely used to describe the morphology evolution of crystal including tow-dimensional material. The formation energy can determine the reactions occurring in which directions and how the edge type was energetically stable. For example, the carbon atoms formed covalent bonds with the atomic step of Ge[$\overline{1 }10$] due to the lattice matching between lattice constant of armchair edge of Graphene and Ge[$\overline{1 }10$] during the growth stage. Therefore, the KWC theory was used to predict the progress of the morphology evolution according to the formation energy. As shown in Fig. 4a, In cases of 0.75 s.c.c.m. and 0 s.c.c.m CH_4_, the zigzag edges of the graphene dots and GNRs showed the higher anisotropic etching rate than armchair edges of the graphene dots and GNRs, which meant the armchair edges had a higher formation energy^53^. On the other hand, in cases of the 1.1–1.4 s.c.c.m. CH_4_, the zigzag edges of the graphene dots and GNRs showed the higher anisotropic growth rate than armchair edges of the graphene dots and GNRs. Eventually, the edge type of the graphene dots and GNRs can be shaped from all kinds of combination of armchair, chiral, and zigzag edges to the thermodynamic favorable armchair edges after regrowth process^54,55^. Due to the anisotropic growth and etch rate of the graphene dots edges on Ge(110) under 0.75 s.c.c.m. and 1.1 s.c.c.m. CH_4_, which was shown in the Fig. 4b, the graphene dots can be thermodynamically shaped to the angular, long, and narrow graphene grain with the included angle of 60° under the growth condition; conversely, the graphene dots can be converged to the energetical favorable hexagonal graphene grain with the included angle of 120° under the etch condition. The schematic diagram of the regrowth process of the edges of the graphene dots was shown in Fig. 4c. Initially, the edges of the graphene dots were in the situation of various zigzag and armchair combinations; after regrowth process, the edges of the graphene dots can be transformed into the thermodynamic favorable armchair edges under both of etch and growth conditions in accordance with the KWC theory. After the sequential experiments of repairing the edge of graphene nanoribbons, the etch rate and the growth rate of graphene along with the different angles confirming that the growth rate and the etch rate was higher at the zigzag edges.

**Figure 5:**
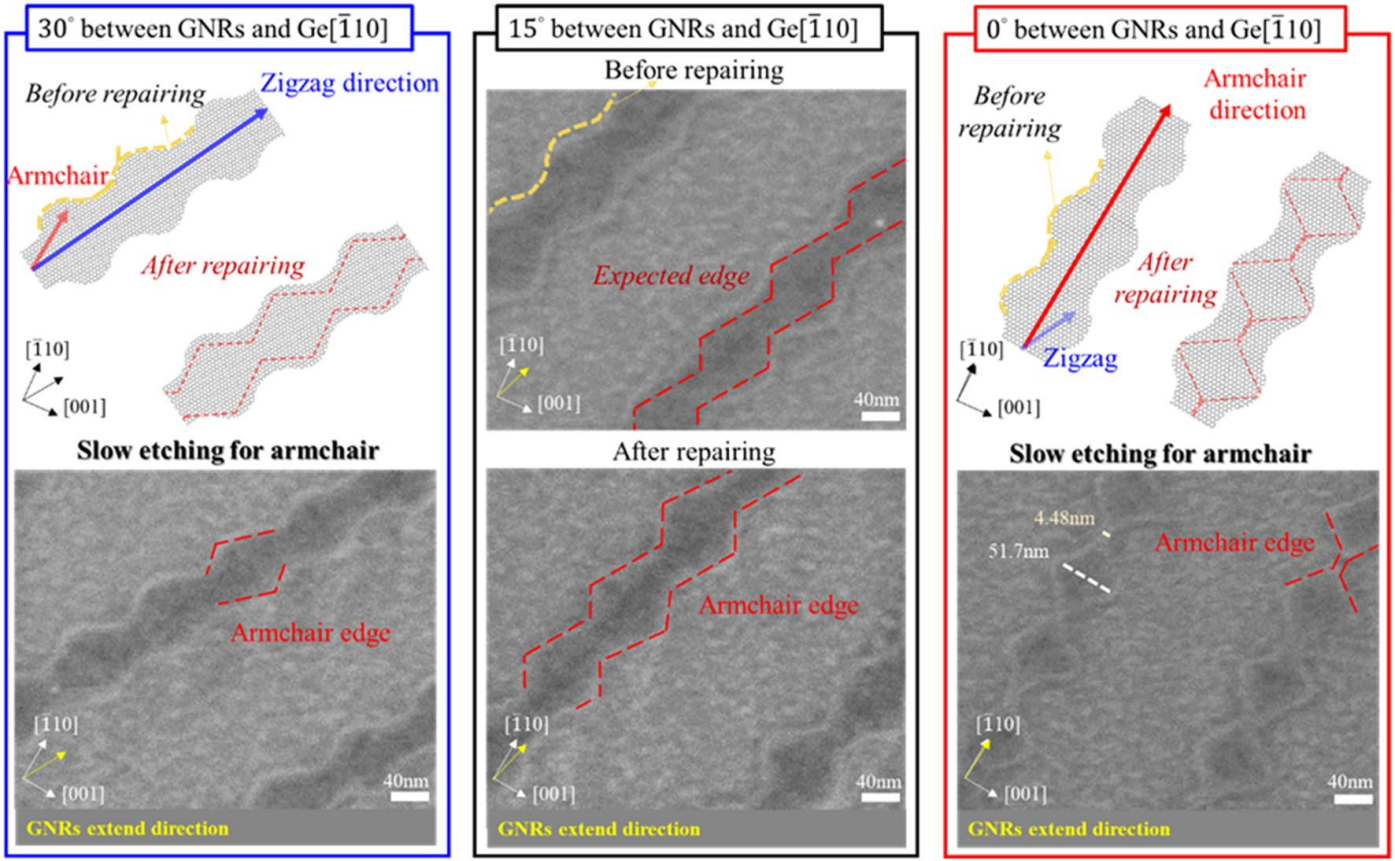
The schematic diagram and SEM images of the CoveGNRs along with along with different directions 0°, 15°, and 30° with respect to Ge[$\overline{1 }10$].

With the optimization of edge regrowth condition and rotation angles of the pre-define GNRs related to Ge[$\overline{1 }10$], the wavy GNRs geometrically transformed to the CoveGNRs structure after regrowth. Here, we compare the wavy structures GNRs with three different angle deviations: 0°, 15°, 30°, from the Ge[$\overline{1 }10$], where armchair edge of graphene is dominant condition as shown in Fig. 5. Based on the angle difference between the GNRs and Ge[$\overline{1 }10$], the different angle of the wavy GNRs were transformed into different edge shapes; meanwhile, the designed wavy structures can assist the edge of GNRs to quickly shrink to the most stable armchair edges. After regrowing under 0.75 s.c.c.m. CH_4_, 200 s.c.c.m. Ar, and 100 s.c.c.m. H_2_ conditions, of which the etching rate of the graphene dots and GNRs was listed in Fig. 4d, the edges of the 30° mismatched between direction of GNRs and Ge[$\overline{1 }10$] would be shaped from the zigzag edges to the most stable armchair edges because the etch rate of zigzag edge was much faster than armchair edge, leading to the convergence of the unstable pentagon- heptagon (5–7) zigzag edges and the pristine zigzag edges to the obtuse parallelogram with the armchair edges according to the KWC theory. The edges of the 15° mismatched between direction of GNRs and Ge[$\overline{1 }10$] would be converged from chiral edges to the armchair edges and the morphology evolution after regrowth would combine the shapes of the CoveGNRs of the 30° mismatched and 0° mismatched cases, forming the morphology transition of the CoveGNRs with the armchair edges. The edges of the 0° mismatched between direction of GNRs and Ge[$\overline{1 }10$] case can be considered as the combination of the unstable 6–7–7 armchair edges, 5–7 armchair edges, 5–6 armchair edges, and pristine armchair edges before the regrowth process^56,57^. After the regrowth process, the same procedure can be adapted to this case, the edges of the GNRs can be energetically converged to the rectangular shape with the most energetical stable pristine armchair edges and reduced the width of GNRs with accurate regrowth process, further producing the waist of the CoveGNRs with width of 4–14 nm on the Ge $(110)$ substrate; meanwhile, based on the tight-binding simulations we calculated., the CoveGNRs with width of 4–14 nm can open the bandgap from 0.1 to 0.3 eV and exclude the zero bandgap caused by the conventional 3N + 2 rule, perfectly enhancing the Graphene-based electronic devices utilization in future.

## Conclusion

We have demonstrated the our designed CoveGNRs can exclude the zero bandgap and can be fabricated through lithography and anisotropic repairing in wafer scale dimension. With considering the electron–phonon scattering rate, the mobility of Cove GNRs is about 2 orders higher compare with the ZGNRs with the width atoms equals to 4. It is found that such structure has better mobility and has larger band gap than ZGNR of the same average width. The band gap of CoveGNRs is about 0.3–0.1 eV in the width range from 4 to 14 nm that is bigger than AGNRs. With the random edge of Cove GNRs from fabrication taken into account, the cove structure exceed 3 nm can converge the variation of bandgap from molecular dynamic in 0.2 ± 0.1 eV when width in 4 nm. The roughness edge of patterned nanoribbons is repaired through "balanced condition growth" in CVD to achieve much precise repairing process. This growing condition can lead an anisotropic annealing to form the regular cove structure in armchair edge along the GNRs with single-crystalline and single-orientation on Ge (110). This method can improve the electrical properties of GNRs because it is highly depending on the defect and edge quality of the materials. The balanced condition growth makes the cove shape sharper and enhance the mobility with open bad gap, and CoveGNRs can become a material that can be used in the semiconductor industry.

## Supplementary Information

Supplementary Information.

## Data Availability

The datasets used in this article available from the corresponding author upon reasonable request.
