# Supplementary material for: Electron–phonon interaction toward engineering carrier mobility of periodic edge structured graphene nanoribbons
Source: Sci Rep. 2023 Apr 8;13:5781. doi: 10.1038/s41598-023-32655-9 (PMC10082836; doi:10.1038/s41598-023-32655-9)
Supplement: Supplementary file 1 — Supplementary Information. [file 41598_2023_32655_MOESM1_ESM.docx]

**Supplementary Information**

Electron-Phonon Interaction toward Engineering Carrier Mobility of Periodic Edge Structured Graphene Nanoribbons

Teng-Chin Hsu^1*^, Bi-Xian Wu^1*^, Rong-Teng Lin^1^, Chia-Jen Chien^1^, Chien-Yu Yeh^1^, Tzu-Hsuan Chang^1^

^1^Graduate Institute of Electronics Engineering (GIEE), National Taiwan University, Taiwan

* Corresponding author. E-mail address: tchang9@ntu.edu.tw

Ideal GNRs are one-dimensional (1D) infinite materials that can be established in a unit cell of periodic system. To study the electronic structure in periodic boundary condition, we employ the Density Functional Theory(DFT) with the self-consistent calculation. The initial state of electron density will be guessed, and iteration will be step by step to converge electron density gradually. In the calculation, the energy $\varepsilon_{i}$ in the previous loop and the calculation result of this time will be compared. If the difference is less than the set convergence value It will end the loop, otherwise it will continue the self-consistent loop.

Fig. S1. Self-consistent loop of Kohn-Sham equation in DFT

The electron and phonon characteristic in the DFT calculations are based on the parameters in the table 1. The vacuum layer is 15 Å to eliminate interlayer interaction. The el-ph coupling were calculated by the EPW package with denser nq and nk.


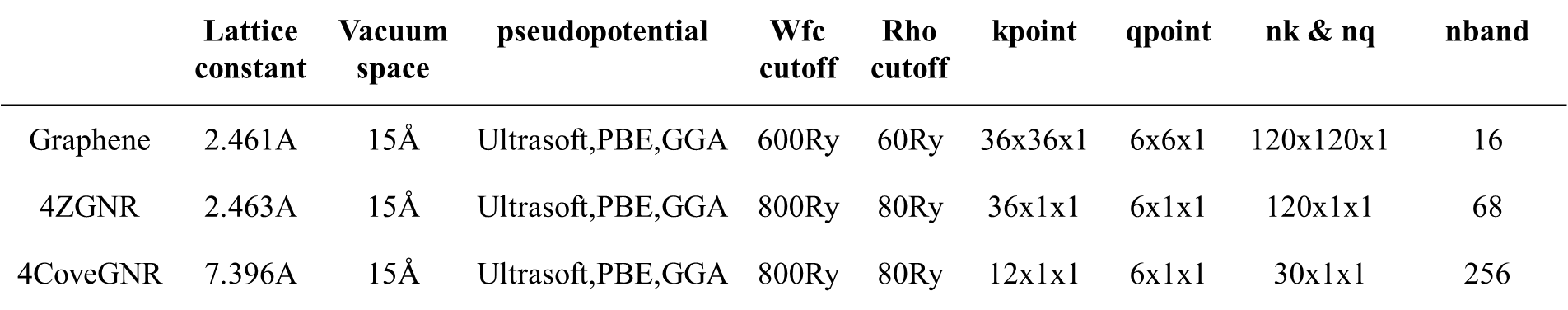


Table. S1. Calculation setting in Quantum Espresso for Graphene, 4ZGNRs and 4CoveGNRs

Here we present the bond length variation of 4ZGNRs and 4CoveGNRs after the structural optimization to reach the equilibrium state. The bond length variation in Figures S2 (a)(b) is nonlinearly distributed throughout the crystal structure. The range of bond length variation is limited to within 2.5%. In both GNRs, a large bond length change can be found at the edges and relatively small changes in the interior. In the case of 4ZGNRs, the alternation of bond length changes is clearly visible, which has an obvious bond length shrinkage at the edges. For 4CoveGNRs, the change of bond length is mainly distributed at the edge, and the bond length does not show alternate changes in the inner region and the range of all changes is limited within 0.5%. It can be found that the edge structure of GNRs with coved shape is compared with ZGNRs. can help the intermediate regions of GNRs not be affected by deformation.


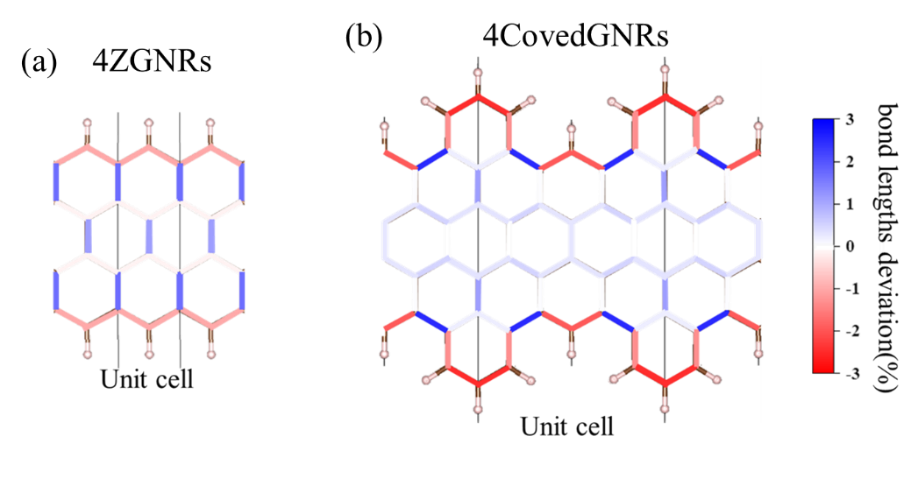


Fig. S2. The lattice structure of graphene ribbons with cell relaxation (a) 4ZGNR (b) 4CoveGNRs. The ratio of bond lengths represents the deviation from the graphene value, 1.421 Å, in percentage.

The band diagram of 4ZGNRs and 4CoveGNRs in DFT method was calculated. The CBM and VBM of 4ZGNRs are overlap to close the bandgap because of the localization on the edge of the wavefunction distribution of CBM and VBM. However, CBM /VBM of CoveGNRs appears in different positions of ZGNRs. The CBM and VBM of the original ZGNRs are at the high symmetry point X in reciprocal k space. With the lattice constant of 4CoveGNRs three times larger than 4ZGNRs, the reciprocal k space of 4CoveGNRs will be reduced to 1/3, and the CBM and VBM originally at the X point will be folded to the Γ point.


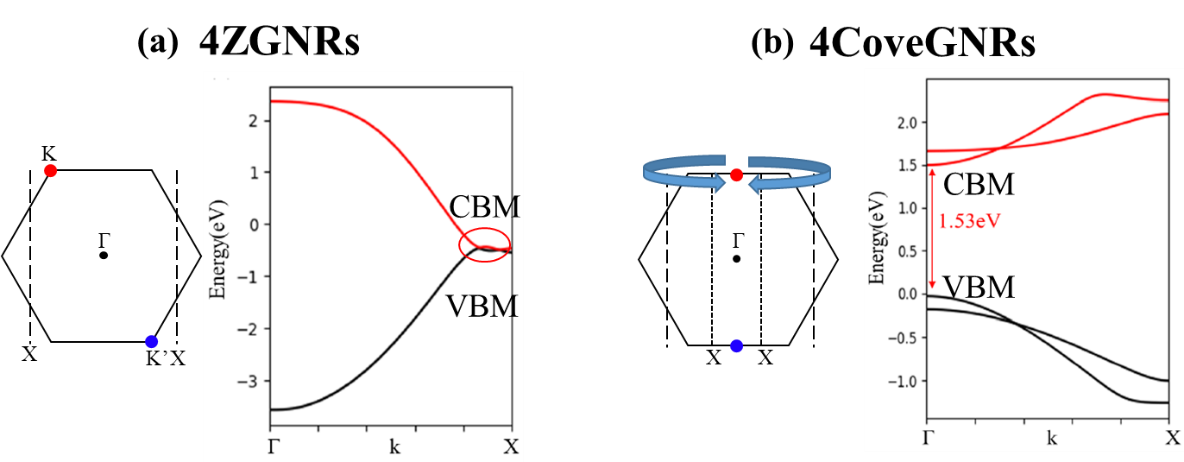


Fig. S3. Reciprocal k space and band diagram of (a)4ZGNRs and 4CoveGNRs.

To reduce the calculation resource of the electron phonon interaction, the electronic band structures in DFT calculations were extracted by Wannier interpolation and compared with the band structures from DFT calculation in coarse k-grid(36x1x1 for 4ZGNR and 12x1x1 for 4CovedGNRs). The Wannier interpolation is to convert the electronic Hamiltonian in plane wave basis under QE calculation into the tight binding Hamiltonian based in maximum localized wannier functions(MLWFs) basis. As we know that the tight-binding Hamiltonian with LCAO can simplify and effectively describe the electronic structure compare with plane waves a large number of superposition in PAW. With Wannier interpolation in MLWFs, subsequent computations such as phonon dispersion or el-ph coupling can be practical in computation time.


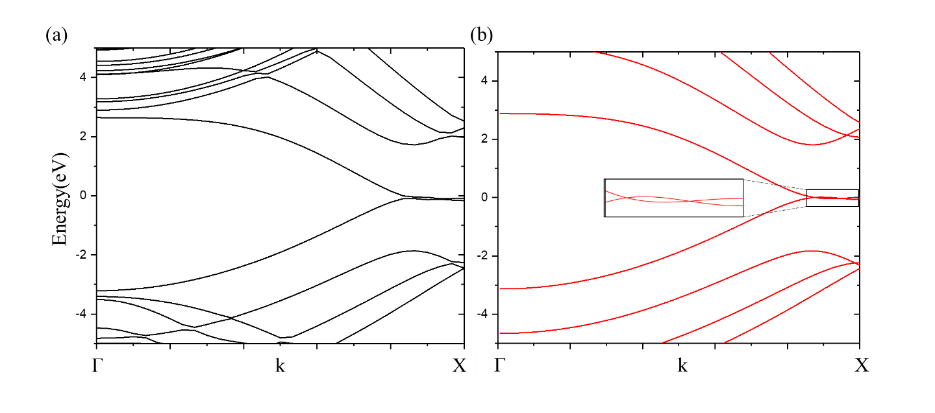


Figure S4. The band structures for 4ZGNRs along the high symmetry lines from Г to X by (a) DFT calculations and (b) Wannier-interpolations


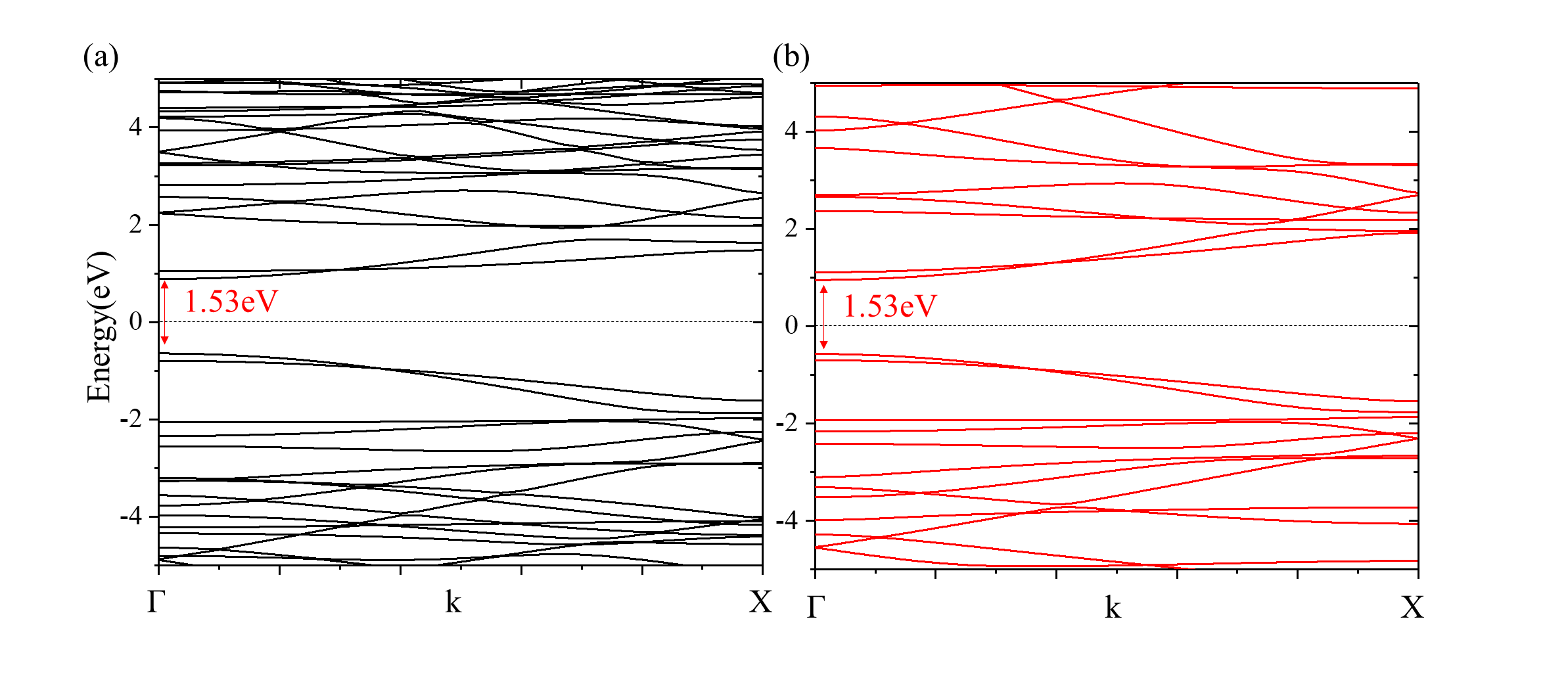


Fig. S5. The band structures for 4CovedGNRs along the high symmetry lines from Гto X by (a) DFT calculations and (b) Wannier-interpolations

To ensure that the transformation from PAW substrates to MLWFs substrates satisfies the locality of the elements of the electron Hamiltonian, phonon dynamics matrix, and el-ph matrix in real space, we examine the spatial distribution of these quantities in 4ZGNRs and 4CovedGNRs. These physical quantities all have a decisive influence on the subsequent calculation of el-ph interaction, so it is necessary to ensure that the electronic structure and phonon dispersion calculated by DFT can be completely transformed into the wantnier interpolation without distortion.


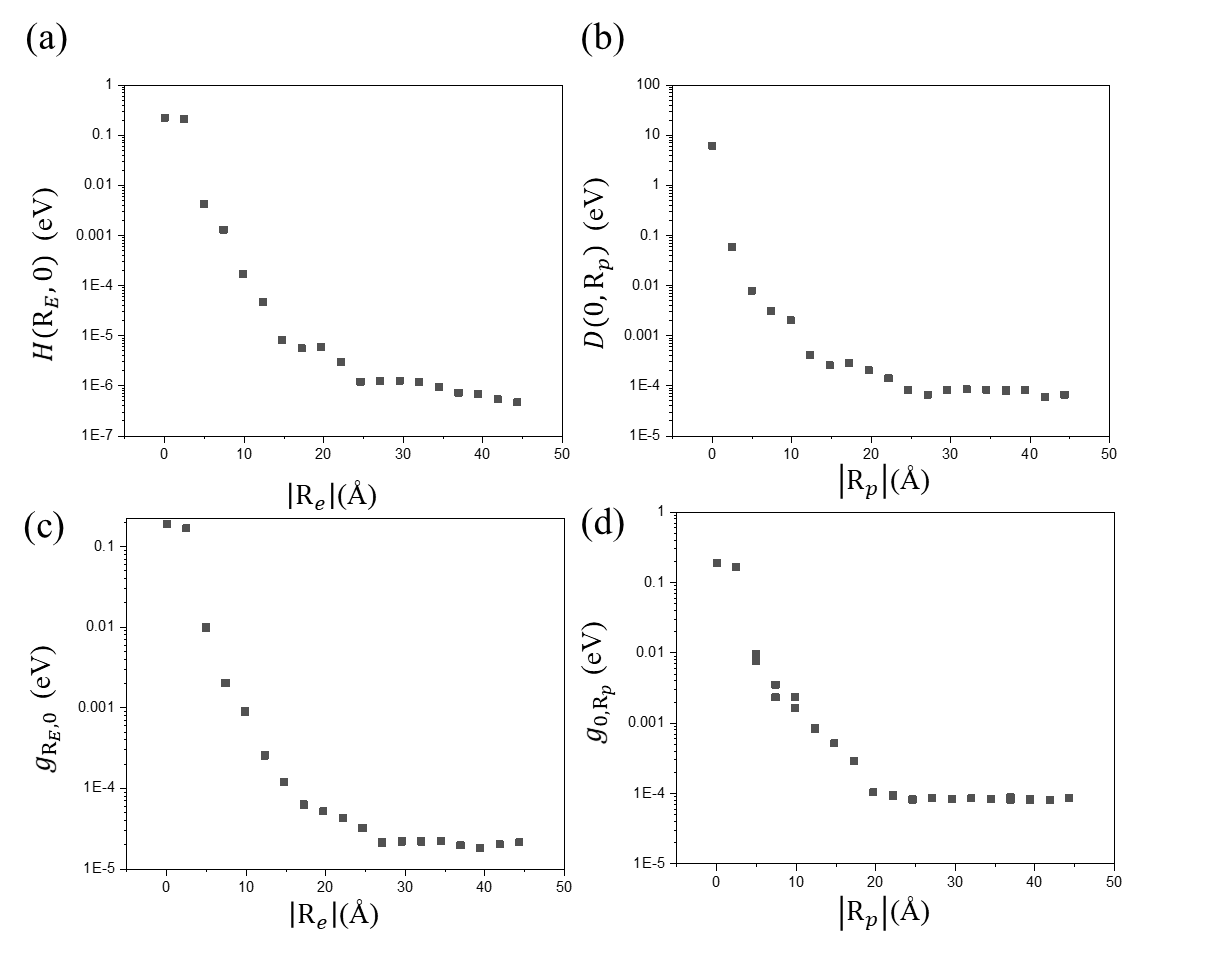


Fig. S6. The electron phonon interaction of 4ZGNRs convergence along with the spatial extension. (a) the Hamiltonian $H_{R_{e}}^{el}$, (b) the dynamical matrix $D_{R_{p}}^{ph}$, (c) the electron phonon coupling matrix elements $g_{R_{e}}$ along the $R_{e}$ (d) the electron phonon coupling matrix elements $g_{R_{p}}$ along the $R_{p}$.


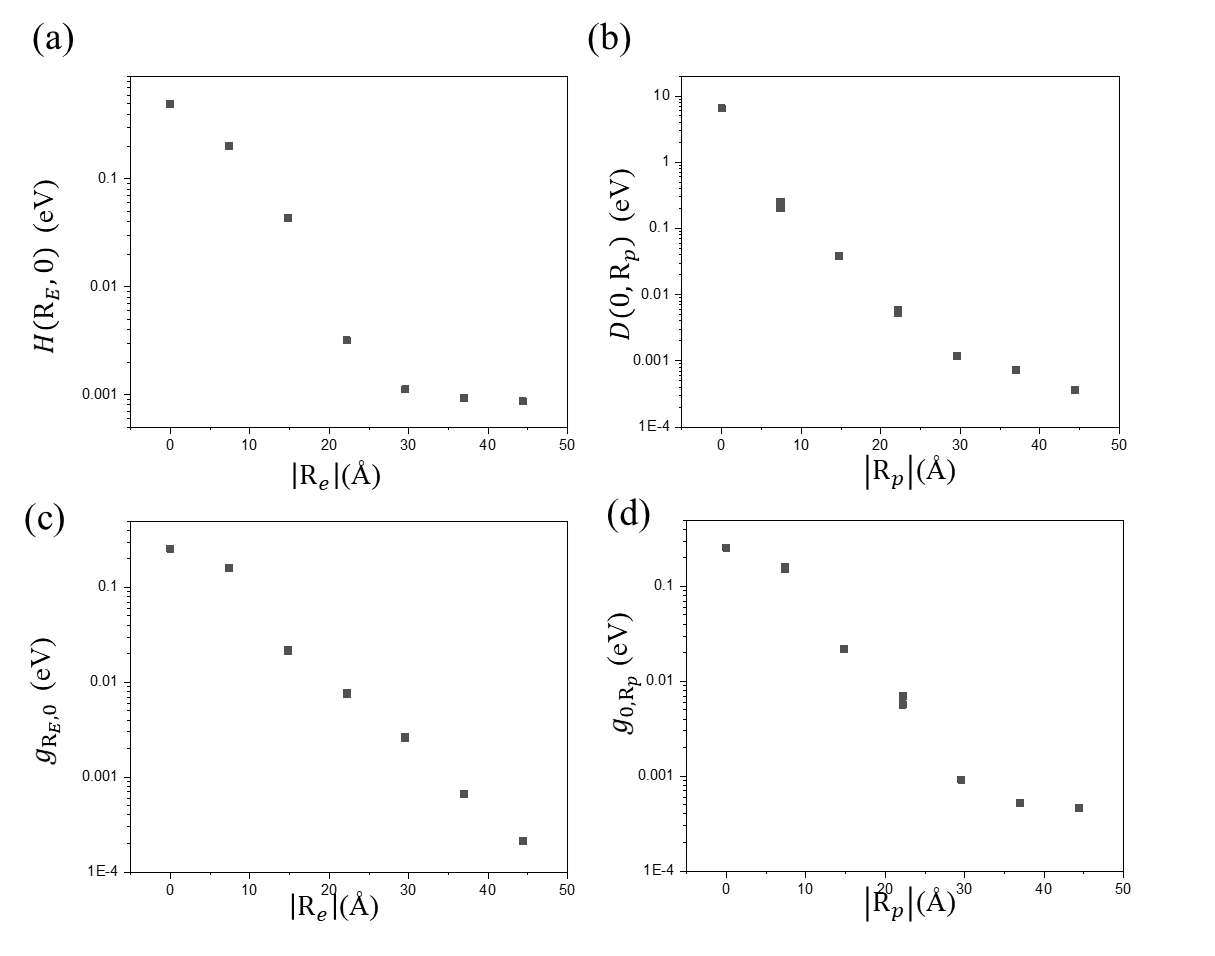


Fig. S7. The electron phonon interaction of 4CoveGNRs convergence along with the spatial extension. (a) the Hamiltonian $H_{R_{e}}^{el}$, (b) the dynamical matrix $D_{R_{p}}^{ph}$, (c) the electron phonon coupling matrix elements $g_{R_{e}}$ along the $R_{e}$ (d) the electron phonon coupling matrix elements $g_{R_{p}}$ along the $R_{p}$ .

We use the number of atoms along the width of the ribbon (*n*) and the number of atoms of cove shape in height (*m*) to define the geometry of CovedGNRs. Besides, to consider the influence of edge roughness in CoveGNRs to the bandgap, we design the our proposed structures with *d,t,l,*θ these parameter, in Fig. S8.


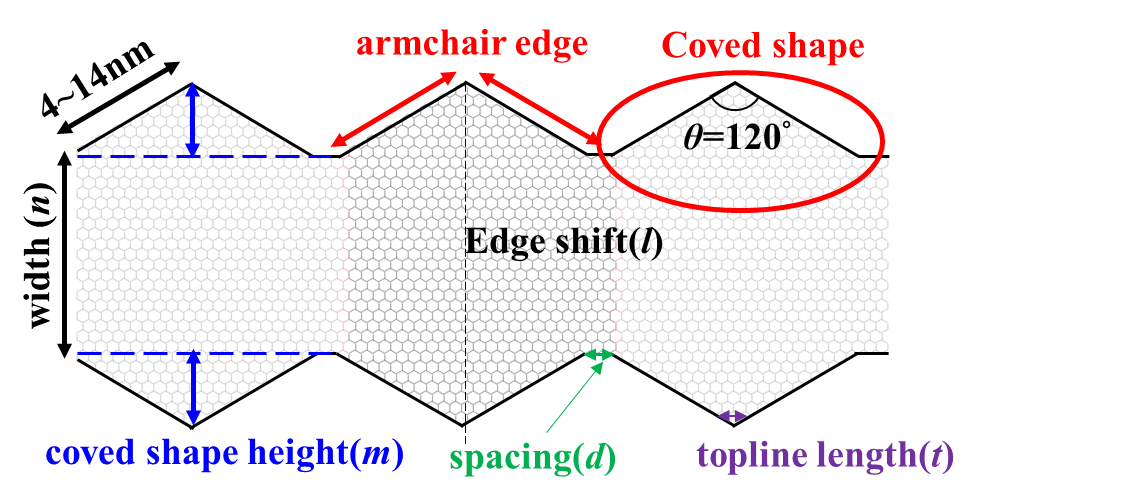


Fig. S8. Schematic of designed n-m-Coved-GNRs-t-l-d. 6-CovedGNRs-10-2-3 for example in this scheme.

The bandgap of CoveGNRs and its width are in negative correlation. There are two kind of trend of bandgap of CoveGNRs as function of its width, the atoms along the width(*n*) equals to even or odd. The trend of bandgap of CoveGNRs are unlike the case of AGNRs with three trend. The bandgap of CoveGNRs decreases as the number of atoms of cove structure in height (*m*), and this reduction is lesser when the width (*n*) of GNRs is broader.


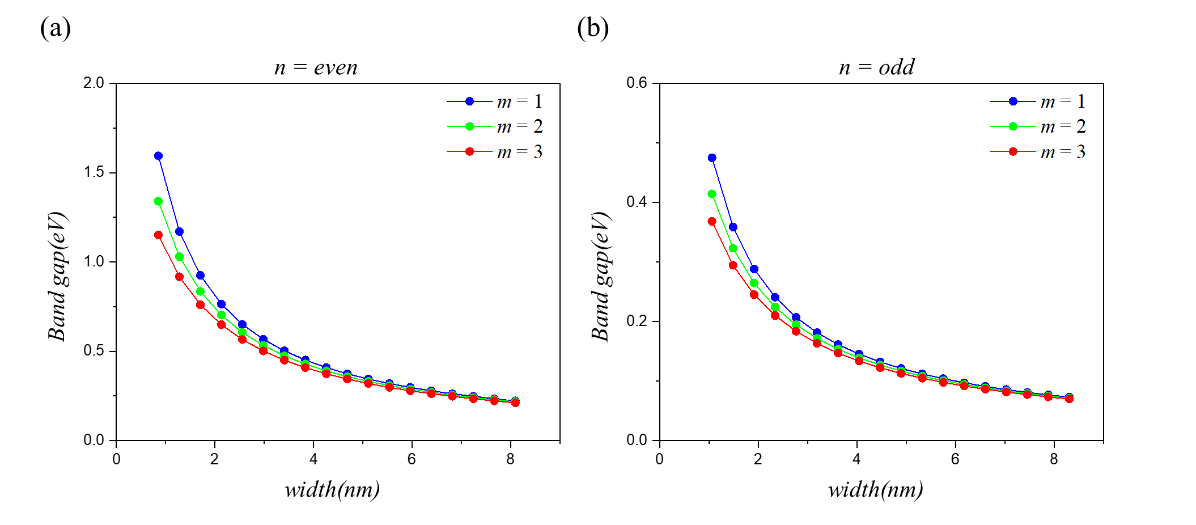


Fig. S9. The bandgap of CoveGNRs as function of its width under *m*=1,2,3.(a) *n*=even and (b) *n* =odd

The formation mechanism of zigzag in the topline of cove structure is due to the fact there is no enough space to form the armchair edge when *t* equal to 0 ~ 2. When *t* equal to 3, there have enough space to form armchair edge on the topline like in case *t* equal to 0. In addition, since different amounts of zigzag segments will change the boundary condition of the wavefunction in CoveGNRs, the band structures of different *t* are non-similar and lead to change in the bandgap. Even though, there are three trend of band gap in CoveGNRs with differnet *t* as a function of cove shape height. In the case of CoveGNRs in width 4.26nm, the band gap close to 0 in the case *t* equal to 1,2 when the height of cove shape is smaller than 1 nm. This zero-bandgap condition can be eliminated when the cove shape becomes bigger to reach the lithography availability.


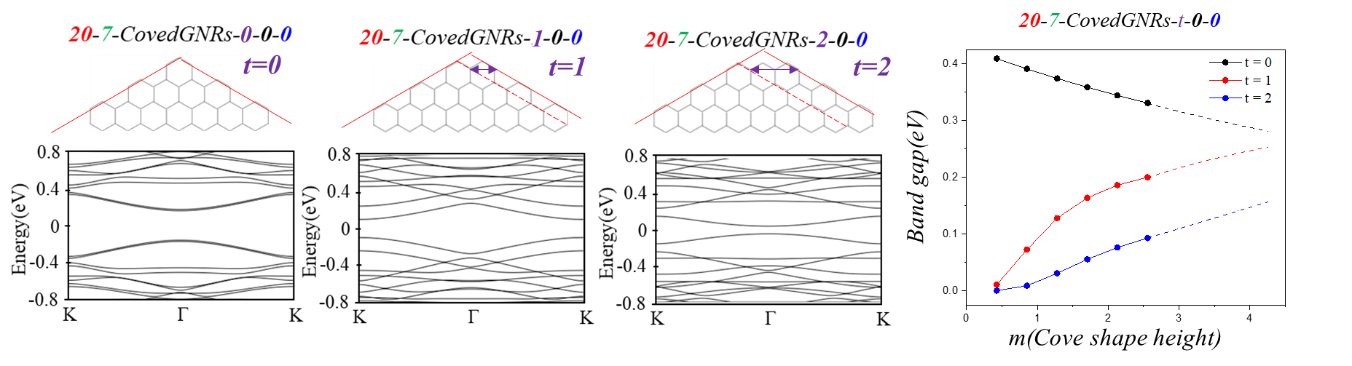


Fig. S10. The lattice structure and band structure of different amount zigzag(*t=0~2*) in topline of cove shape size of CoveGNRs. Bandgap variation versus zigzag edge amount(*t*) in the in topline of cove shape size of Cove GNRs. The cove shape of CoveGNRs increasing can eliminate the zero band gap caused by zigzag edge segment of Cove GNRs.


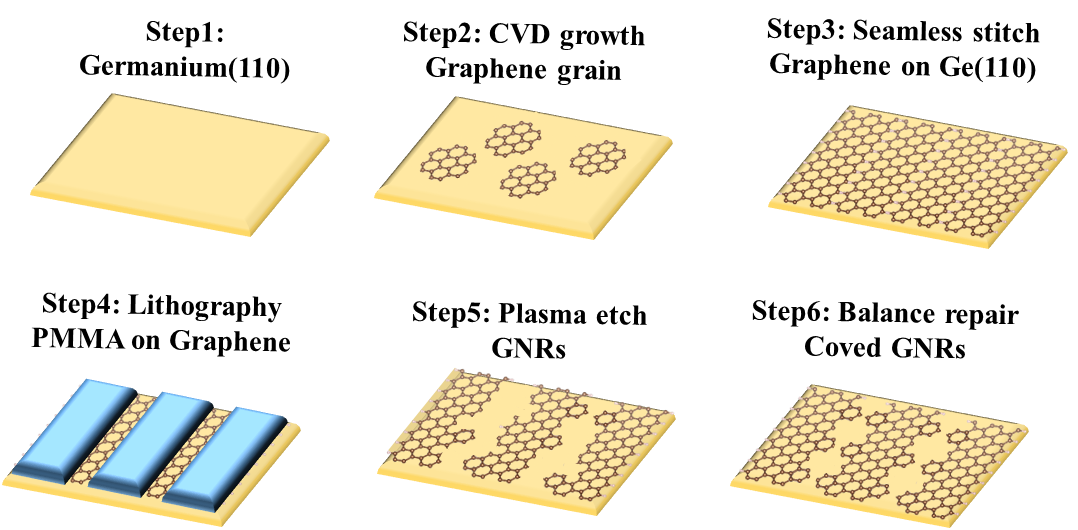


Fig. S11. Process flow of CoveGNRs fabrication. CVD growth graphene on Ge(110) were patterned through lithography and etching and then repair its edge in balance repair condition.


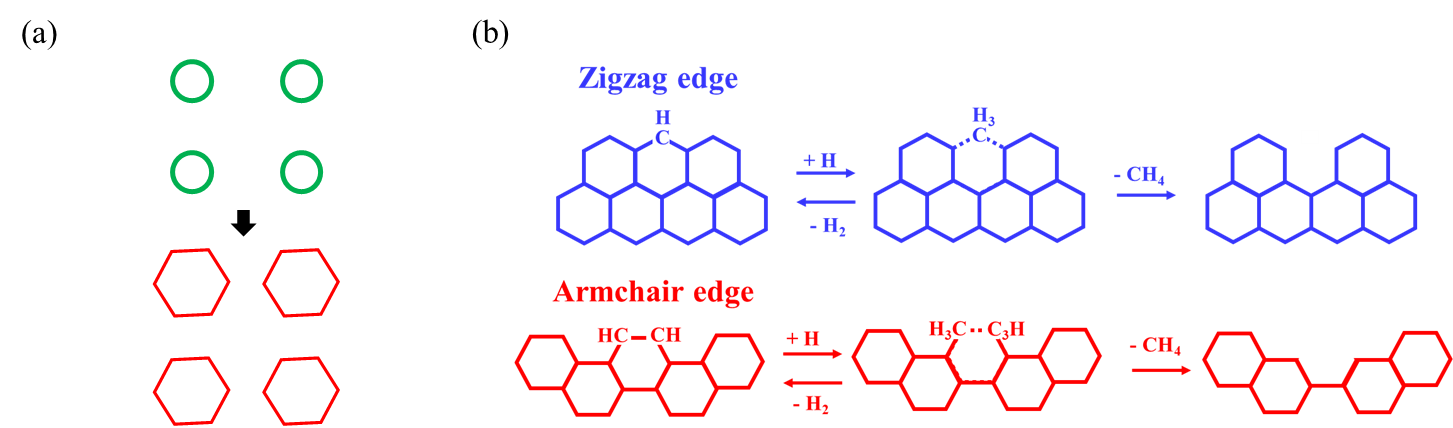


Fig. S12. (a) Schematic to illustrate the anisotropic etching and (b) process of the molecular dynamics in (1) the pi bond at edge of graphene is hydrogenated into a sigma bond with lower potential barrier at high temperature. (2) the carbon bond of adjacent edge carbon atoms are attacked and broken by high-energy hydrogen atoms, resulting in CH_3_ group; (3) The unstable CH_3_ group is volatile, and an etching is completed.


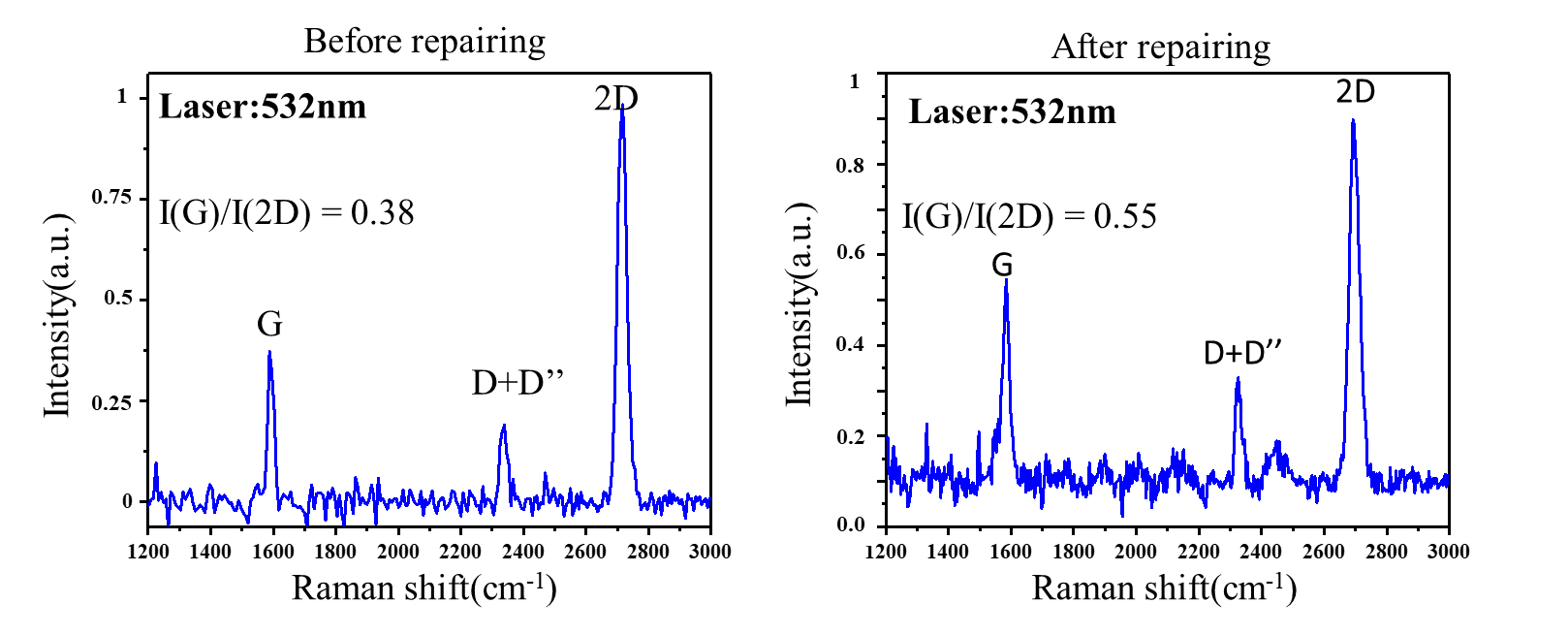


Fig. S13. Raman spectrum of GNRs before and after edge repairing under the equilibrium growth control.


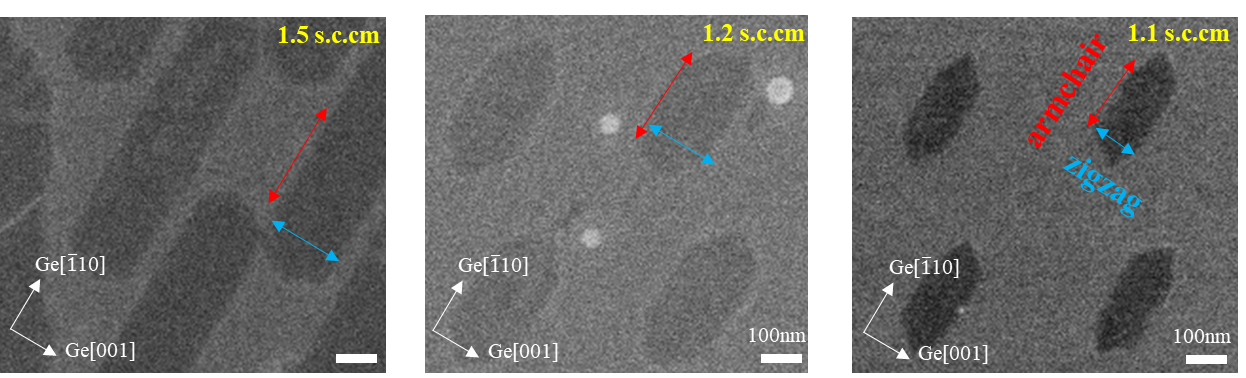


Fig. S14. Fast growing direction along Ge[$\bar{1}10$] of graphene dot in different CH_4_ gas flow rate


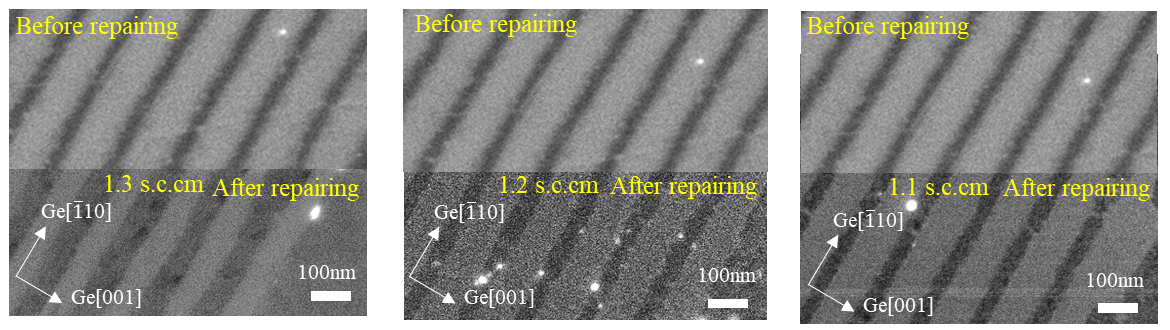


Fig. S15. Different width reduction of GNRs of 0 degree difference between GNRs and Ge[$\bar{1}10$] in 1.1, 1.2, 1.3 s.c.c.m CH_4_ gas flow rate


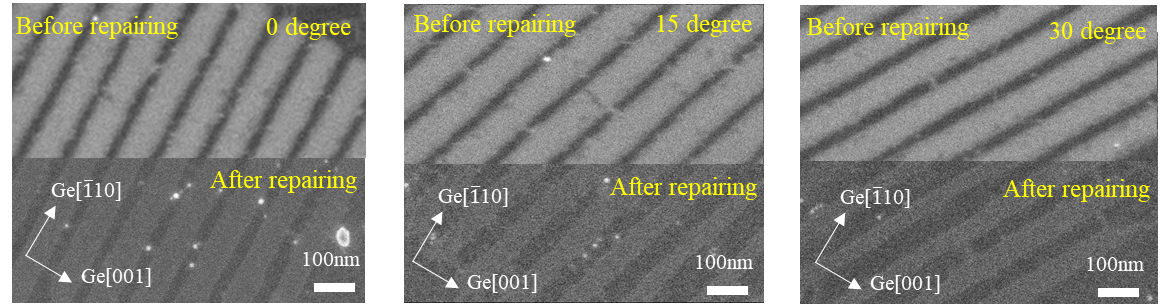


Fig. S16. SEM images of the GNRs along with different directions 0^0^, 15^0^, and 30^0^ with respect to Ge[$\bar{1}10$] in 1.1 s.c.c.m CH_4_ gas flow rate


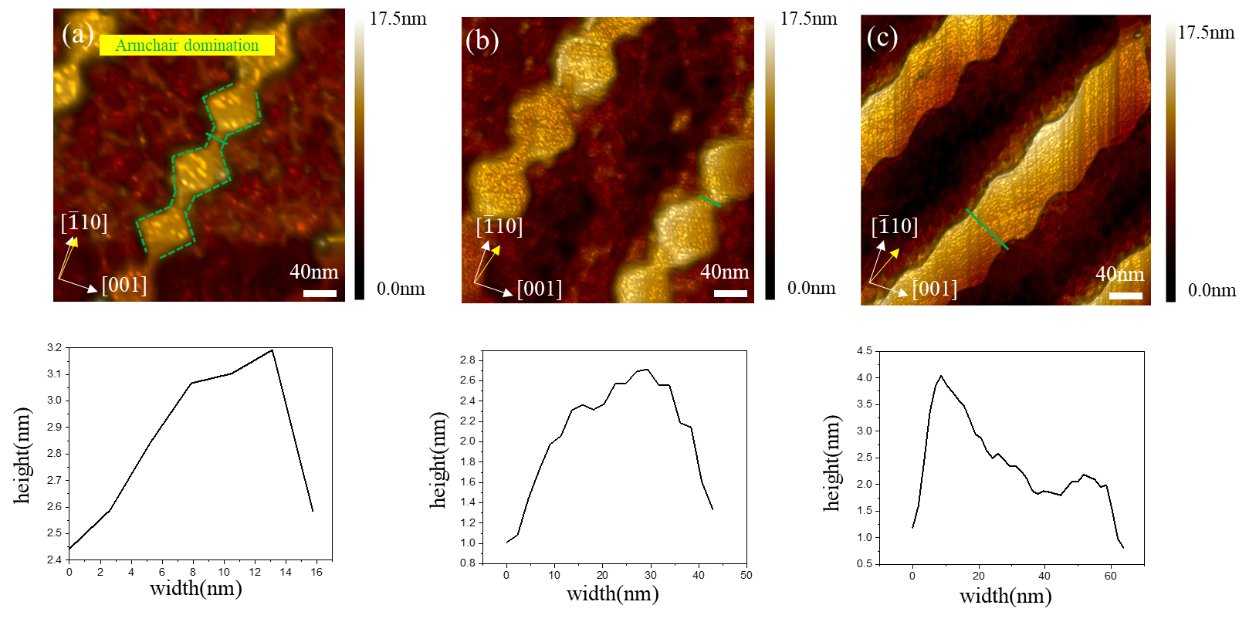
The AFM image of CoveGNRs was investigated by Bruker Dimension Icon microscope operating in ScanAsyst Mode and probe pin is made of Silicon Nitride in tip radius maximum about 12nm in 0.4N/m.

Fig. S17. AFM image and the scale of height label by green solid line of CoveGNRs after edge repairing along with different directions (a)0^0^, (b)15^0^, and (c)30^0^ with respect to Ge[$\bar{1}10$]
